# Future perspectives: Novel Frontiers in Cancer Metastasis

**DOI:** 10.1007/s10585-022-10152-z

**Published:** 2022-02-12

**Authors:** Stanley P. Leong, Jonathan S. Zager

**Affiliations:** 1 California Pacific Medical Center and Research Institute, San Francisco, CA USA; 2 University of California San Francisco School of Medicine, San Francisco, CA USA; 3 Department of Cutaneous Oncology, Moffitt Cancer Center, Tampa, FL USA; 4 Morsani College of Medicine, University of South Florida, Tampa, FL USA

As stated in the Editorial [1], the primary goal of this Special Issue: Novel Frontiers in Cancer Metastasis is to crystalize the concept that cancer is a heterogeneous disease resulting from genomic alterations or mutations. Thus, heterogeneous cellular clones may develop with different biological characteristics. Recent studies have emphasized the importance of the cancer microenvironment, including immune responses, in modulating the growth of these heterogeneous clones. The initial route for cancer spread may be through lymphatic vessels to the sentinel lymph nodes or by way of blood vessels, or via both systems. The molecular mechanisms for these metastatic routes are not well understood. These important characteristics of cancer progression and metastasis have been recently summarized in an upcoming textbook on Cancer Metastasis through the Lymphovascular System [2].

Our international conference on cancer metastasis was inaugurated in 2005 and it has been successfully held biennially with several publications addressing the biology and treatment of cancer metastasis. In 2017, the 7th International Cancer Metastasis Congress through the Lymphovascular System: Biology & Treatment, preceding our last biennial congress in 2019, was held from April 20–22, 2017 (www.cancermetastasis.org). Invited review articles from the presenters were published in a special issue by Clinical and Experimental Metastasis [3]. The 2019 8th International Cancer Metastasis Congress through the Lymphovascular System was held from October 25-27, 2019 (www.cancermetastasis.org). The presentations were written up as review articles to be published in this Special Issue: Novel Frontiers in Cancer Metastasis [1].

In the upcoming 9th International Cancer Metastasis Congress, planned for spring 2023, we will address the latest findings of molecular and genetic mechanisms of cancer heterogeneity, proliferation and metastasis. Molecular biomarkers will be described in detail in relation to the metastatic pathways of cancer via the tumor-associated lymphatic and blood vessels. Further, the effect of cancer microenvironment on the progression of cancer metastasis will be extensively discussed with respect to the stromal cells consisting of fibroblasts, lipocytes, immune cells, lymphatic and blood vessels as well as other parenchymal cells.

The critical role of the immune system within the primary and metastatic tumor will be explored, taking into consideration the concepts and findings of immune checkpoint molecules such as CTLA-4 and PD-1, which are associated with T cell inhibition and cancer growth. Immunotherapy with immune checkpoint blockade has resulted in the activation of cytotoxic T cells and cancer destruction [4, 5]. The sentinel lymph node may serve as an incubator for caner to grow and act as a gateway for systemic metastasis. The molecular interaction between T cells and the cancer proliferation needs to be addressed in detail. While it is important to understand the biology and mechanisms of cancer expansion, it is also important to study the T cell repertoire within the cancer microenvironment, which should be considered as an ever-changing profile in relationship to cancer growth, which is associated with mutation and formation of neoantigens. The overall goal is to understand the molecular mechanisms of cancer metastasis so that therapeutic strategies can be deveoped to block the process of cancer metastasis.

## Data Availability

Data sharing not applicable to this article as no datasets were generated or analyzed during the current commentary.
